# Proposed clinical phases for the improvement of personalized treatment of checkpoint inhibitor–related pneumonitis

**DOI:** 10.3389/fimmu.2022.935779

**Published:** 2022-07-20

**Authors:** Chengzhi Zhou, Yilin Yang, Xinqing Lin, Nianxin Fang, Likun Chen, Juhong Jiang, Haiyi Deng, Yu Deng, Minghui Wan, Guihuan Qiu, Ni Sun, Di Wu, Xiang Long, Changhao Zhong, Xiaohong Xie, Zhanhong Xie, Ming Liu, Ming Ouyang, Yinyin Qin, Francesco Petrella, Alfonso Fiorelli, Sara Bravaccini, Yuki Kataoka, Satoshi Watanabe, Taichiro Goto, Piergiorgio Solli, Hitoshi Igai, Yuichi Saito, Nikolaos Tsoukalas, Takeo Nakada, Shiyue Li, Rongchang Chen

**Affiliations:** ^1^State Key Laboratory of Respiratory Disease, National Clinical Research Centre for Respiratory Disease, First Affiliated Hospital, Guangzhou Institute of Respiratory Health, Guangzhou Medical University, Guangzhou, China; ^2^Affiliated Dongguan People’s Hospital, Dongguan Institute of Respiratory and Critical Care Medicine, Southern Medical University, Dongguan, China; ^3^Department of Medical Oncology, State Key Laboratory of Oncology in South China, Collaborative Innovation Center for Cancer Medicine, Sun Yat-sen University Cancer Center, Guangzhou, China; ^4^Shenzhen People’s Hospital, The Second Clinical Medical College of Jinan University, The First Affiliated Hospital, Southern University of Science and Technology, Shenzhen, China; ^5^Department of Respiratory Disease, Peking University Shenzhen Hospital, Shenzhen, China; ^6^Division of Thoracic Surgery, European Institute of Oncology, IRCCS, Milan, Italy; ^7^Department of Oncology and Hemato-oncology, University of Milan, Milan, Italy; ^8^Thoracic Surgery Unit, University of Campania Luigi Vanvitelli, Naples, Italy; ^9^IRCCS Istituto Romagnolo per lo Studio dei Tumori (IRST) "Dino Amadori", Meldola, Italy; ^10^Department of Internal Medicine, Kyoto Min-Iren Asukai Hospital, Kyoto, Japan; ^11^Department of Respiratory Medicine and Infectious Diseases, Niigata University Graduate School of Medical and Dental Sciences, Niigata, Japan; ^12^Lung Cancer and Respiratory Disease Center, Yamanashi Central Hospital, Yamanashi, Japan; ^13^Division of Thoracic Surgery & Lung Transplantation, IRCCS Azienda Ospedaliero-Universitaria di Bologna, Bologna, Italy; ^14^Department of General Thoracic Surgery, Japanese Red Cross Maebashi Hospital, Maebashi, Japan; ^15^Department of Surgery, Teikyo University School of Medicine, Tokyo, Japan; ^16^Department of Oncology, 401 General Military Hospital, Athens, Greece; ^17^Division of Thoracic Surgery, Department of Surgery, The Jikei University School of Medicine, Tokyo, Japan

**Keywords:** immune checkpoint inhibitor, checkpoint inhibitor-related pneumonitis, clinical phases, management, glucocorticoids

## Abstract

**Background:**

Checkpoint inhibitor–related pneumonitis (CIP) is a lethal immune-related adverse event. However, the development process of CIP, which may provide insight into more effective management, has not been extensively examined.

**Methods:**

We conducted a multicenter retrospective analysis of 56 patients who developed CIP. Clinical characteristics, radiological features, histologic features, and laboratory tests were analyzed. After a comprehensive analysis, we proposed acute, subacute, and chronic phases of CIP and summarized each phase’s characteristics.

**Results:**

There were 51 patients in the acute phase, 22 in the subacute phase, and 11 in the chronic phase. The median interval time from the beginning of CIP to the different phases was calculated (acute phase: ≤4.9 weeks; subacute phase: 4.9~13.1 weeks; and chronic phase: ≥13.1 weeks). The symptoms relieved from the acute phase to the chronic phase, and the CIP grade and Performance Status score decreased (*P*<0.05). The main change in radiologic features was the absorption of the lesions, and 3 (3/11) patients in the chronic phase had persistent traction bronchiectasis. For histologic features, most patients had acute fibrinous pneumonitis in the acute phase (5/8), and most had organizing pneumonia in the subacute phase (5/6). Other histologic changes advanced over time, with the lesions entering a state of fibrosis. Moreover, the levels of interleukin-6, interleukin-10 and high-sensitivity C-reactive protein (hsCRP) increased in the acute phase and decreased as CIP progressed (IL-6: 17.9 vs. 9.8 vs. 5.7, P=0.018; IL-10: 4.6 vs 3.0 vs. 2.0, P=0.041; hsCRP: 88.2 vs. 19.4 vs. 14.4, P=0.005).

**Conclusions:**

The general development process of CIP can be divided into acute, subacute, and chronic phases, upon which a better management strategy might be based devised.

## Introduction

Immune checkpoint inhibitors (ICIs) represent a significant breakthrough in the treatment of cancers; however, immune-related adverse events (irAEs) have restricted the application of ICIs. Checkpoint inhibitor–related pneumonitis (CIP) is one of the most common types of severe irAEs. In clinical trials, its incidence is generally at 3–5% (1–3). Nevertheless, a higher incidence ranging from 9.5–19.0% has been disclosed in real-world studies (4–6). Although many studies suggest that irAEs may be related to better efficacy of immunotherapy, the relevant research shows a shorter overall survival in patients with CIP (7, 8). The current treatment of CIP involves glucocorticoid therapy supplemented by immunosuppressive agents (such as infliximab and mycophenolate mofetil) and other supportive therapies as needed (9–13). Moreover, according to clinical experience and investigation, we recently proposed new clinical types of CIP, including the pure type, induced type, and mixed type, which may provide more individual management strategies for patients with CIP (14).

Most related studies have classified CIP and provided suggestions on treatment based on grades, but no studies have focused on the occurrence and development of CIP longitudinally. According to the sequence of histologic changes, the clinical picture of radiation-induced lung injury (RILI) is divided into 3 phases: the early phase, the intermediate phase (acute pneumonitis), and the late phase (pulmonary fibrosis) (15). In many lung diseases, such as hypersensitivity pneumonitis (HP) and RILI, the classical change from inflammation to fibrosis is observed (16). Referring to this well-attested change of lung injury and our own clinical experience, we speculate that CIP proceeds in a similar fashion.

Furthermore, many specific problems regarding the management of CIP remain to be solved: the optimal timing, dose, and duration of treatment with steroids; the potential adverse impact on the increased risk of infection when steroids are used; whether other treatments can be used for better management of CIP and other issues (17). Among them, the duration time of treatment with glucocorticoids for CIP requires attention. Although the relevant guidelines recommend tapering glucocorticoids over 4–6 weeks for grade 2 CIP or ≥6 weeks for grade ≥3 CIP, the time for cessation is not clear. The side effects of long-term usage of glucocorticoids are well-known and notably include infection, which may be one of the critical factors for the poor prognosis and prolonged recovery of CIP patients (18, 19). Moreover, for fibrosis changes, glucocorticoids are no longer considered necessary for treatment (15). Given these two factors, the relative harm versus benefit of glucocorticoid use in the late phase of CIP warrants further investigation.

In this study, we assumed the acute, subacute, and chronic phases of CIP; verified the differences between these proposed phases; and summarized the characteristics of each phase in terms of clinical characteristics, radiological features, histological features, and laboratory tests. Based on these proposed phases, we attempted to provide recommendations for the personalized management of patients with CIP.

## Materials and methods

### Patients

We retrospectively reviewed the clinical data of cancer patients diagnosed with CIP between January 2016 and February 2021 at four different institutions (center 1: First Affiliated Hospital of Guangzhou Medical University; center 2: Collaborative Innovation Center for Cancer Medicine; center 3: Dongguan People’s Hospital; center 4: Shenzhen People’s Hospital). Patients were excluded if they lacked baseline imaging information and critical analysis data. This study was approved by the Institutional Review Board of the First Affiliated Hospital of Guangzhou Medical University (Guangzhou, Guangdong, China, No. 2020-95) and then approved by each participating center.

### Diagnosis of CIP

The diagnosis and differential diagnosis of CIP followed the guidelines of the National Comprehensive Cancer Network, the American Society for Clinical Oncology, the Society for Immunotherapy of Cancer, and the European Society for Medical Oncology (diagnosed by C Zhou and X Lin) (9–12). The diagnosis criteria were the following (1): medication history of ICIs (2); presence of new/worsening symptoms including cough, shortness of breath, with or without fever, and others (3); new primary lesions attributable to ICIs in radiologic images (4); exclusion of other diagnoses, including bacterial pneumonia, tuberculosis, tumor progression and others, basing on laboratory and/or histology tests (however, when the anti-infection therapy was ineffective, CIP coinfection was permitted). Criterion (2) could be absent when the patients were diagnosed with grade 1 CIP. All CIP patients were graded according to Common Terminology Criteria for Adverse Events (CTCAE) version 5.0. If there was controversy, a multi-disciplinary team would be involved in the discussion.

### Data collection

Baseline information of included patients was extracted and had age, sex, smoking history, previous lung diseases, histologic types, tumor–node–metastasis (TNM) stage, history of lung radiation therapy, therapy lines of ICIs, and strategies of ICIs. The TNM stage was classified according to the eighth edition classification of the corresponding tumor. The following data during the CIP period were collected and used for analysis: the date of occurrence, clinical features, Eastern Cooperative Oncology Group Performance Status score, CIP grade, high-resolution computed tomography (HRCT) images, histologic features, and relevant laboratory examination results. The results of HRCT images were individually read by a radiologist (Y Deng) and checked by other authors. The basic histologic features were mainly obtained from routine hematoxylin and eosin (HE) staining. Special stains included phosphotungstic acid hematoxylin staining for fibrinous exudation and Masson’s Trichrome staining for collagen fibers. All histology results were individually reviewed by a pathologist (JH Jiang) and checked by other authors. Laboratory examination included routine blood test, cytokines, krebs von den lungen-6, albumin, gamma-glutamyl transferase, lactate dehydrogenase, high sensitivity C-reactive protein (hsCRP), and procalcitonin.

### Clinical phases of CIP

Clinical phases were preliminarily separated based on disease process, radiologic features (including X-ray and CT), and histologic features (1). The acute phase was defined as a status dominated by inflammation and exudation. When a patient is close to onset (usually within two months), the imaging and histological changes are mainly inflammatory changes (such as ground-glass opacities and consolidation in imaging and cellulose exudation in histology), and the absorption rate of the lesion after treatment is relatively high, we consider this to be the acute phase (2). The chronic phase occurs further in time after onset, with imaging and histology dominated by chronic changes (such as fibrosis changes and traction bronchiectasis in the image and fibrous tissue hyperplasia in histology), and the absorption rate of the lesion is slow or unchanged for an extended period (3). The subacute phase is situated between these 2 phases and has characteristics of both the acute and chronic phases. The time between imaging reviews for CIP patients should not exceed 1.5 months, otherwise the follow-up information is considered insufficient for initially phasing.

Based on this preliminary classification, the continuous information of clinical features, computed tomography (CT) features, histologic features, and laboratory results of the same patient were divided into different periods for further analysis to verify the difference among different phases and discern the characteristics of each phase. When there are two or greater than two information in the same phase, the one closest to the beginning of the phase is selected. The most acceptable distant of the information after the start of the phase is no more than 2 weeks, otherwise, it is considered as the absence of relevant values.

### Statistical analysis

The data were expressed as numbers and percentages for categorical variables and median (minimum to maximum) or median (interquartile range) for continuous variables. The chi-squared or Fisher exact test was used to compare categorical variables, and the Kruskal-Wallis test was used for continuous variables. If the difference was significant, Bonferroni correction was used for pairwise comparison. A 2-sided *P* value of less than 0.05 (*P*<0.05) was considered statistically significant. Missing data were handled by deleting missing cases in the specific analysis. Statistical analyses were conducted using SPSS version 25.0 (IBM Corporation, Armonk, NY, USA). At the same time, data were visualized with GraphPad Prism version 8.0.0 for Windows (GraphPad Software, San Diego, California, USA) and OriginPro, Version 2021 (OriginLab Corporation, Northampton, MA, USA).

## Results

### Patient characteristics

A total of 85 patients (center 1: N=76; center 2: N=5; center 3: N=3; center 4: N=1) were diagnosed with CIP between January 2016 and February 2021. After carefully reviewing patients’ clinical information, 56 patients (center 1: N=48; center 2: N=4; center 3: N=3; center 4: N=1) were included in this analysis (12 without detailed baseline CT information and 17 without enough detailed data to be analyzed). The median age of the included patients was 66 (range: 36–85), 85.7% of patients were male, 7 (12.5%) patients had previous pulmonary disease, and 11 (19.6%) patients had a history of lung radiation therapy. Forty patients (71.4%) received combination therapy. More detailed information on patients’ characteristics before being diagnosed with CIP is shown in **Table 1**. The median follow-up after CIP occurrence was 20.0 (95% CI: 16.1–23.9) weeks.

**Table 1:** The baseline information of patients before diagnosis with checkpoint inhibitor-related pneumonitis (CIP).

Characteristics	Patients (n=56)
Age – y	
Median	63
Range	36-85
Male – N (%)	48 (85.7%)
Female – N (%)	8 (14.3%)
Smoking status – N (%)	
Former/Current	32 (57.1%)
Never	24 (42.9%)
Previous lung disease ^a^ – N (%)	7 (12.5%)
Histologic types – N (%)	
Squamous	24 (42.9%)
Adenocarcinoma	12 (21.4%)
Unclassified NSCLC	1 (1.8%)
Small cell lung cancer	9 (16.1%)
Large cell neuroendocrine tumor	2 (3.6%)
Sarcomatoid carcinoma	1 (1.8%)
Other than lung cancer ^b^	7 (12.5%)
TNM phases – N (%)	
III	21 (37.5%)
IV	27 (48.2%)
Unknown	8 (14.3%)
History of lung radiation therapy – N (%)	11 (19.6%)
Therapy lines of ICIs – N (%)	
First	39 (69.6%)
Subsequent	17 (30.4%)
ICI strategy – N (%)	
Monotherapy	16 (28.6%)
Combination therapy	40 (71.4%)
Immunchemotherapy	31 (77.5%)
ICI+anti-angiogenesis	1 (2.5%)
Immunchemotherapy+anti-angiogenesis	8 (20.0%)
Anti-PD-1/PD-L1 inhibitor– N (%)	
anti-PD-1 inhibitor	53 (94.6%)
anti-PD-L1 inhibitor	3 (5.4%)

y, year; N, number of cases; NSCLC, non-small cell lung cancer; TNM, tumor–node–metastasis; ICI, immune checkpoint inhibitor.

a Previous lung disease includes chronic obstructive pulmonary disease, emphysema, and interstitial lung disease.

b Other histologic types include lung metastatic urothelial carcinoma (N=1), esophageal cancer (N=2), hypopharyngeal cancer (N=1), hepatocellular carcinoma (N=1), colon cancer (N=1) and endometrial carcinoma (N=1).

**Figure 1:**
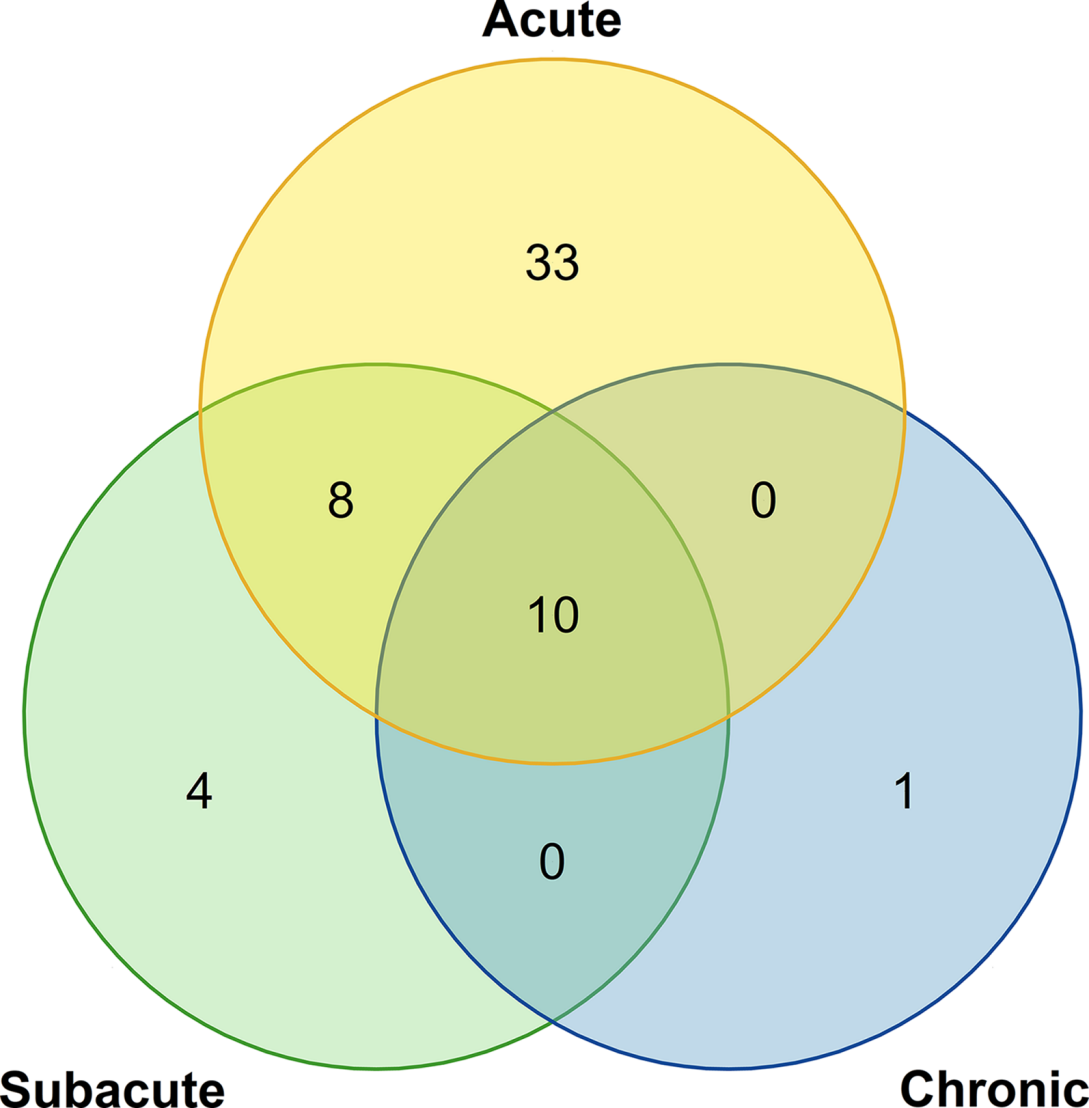
The Venn diagram of the numbers of patients in acute, subacute, and chronic phases.

After preliminary estimation, 51 patients experienced acute phase, 22 experienced subacute phase, and 11 experienced the chronic phase. Among them, four patients were in the subacute phase, and one patient was in the chronic phase before being admitted to one of the four institutions mentioned above (**Figure 1**). All patients were treated based on grade according to the major guidelines. Grade 1 patients were given observation. Grade 2 patients were given 1 to 2 mg/kg/d glucocorticoids, and grade 3-4 patients were given 2 mg/kg/d glucocorticoids. None of the patients included in this study were treated with other immunosuppressive agents.

### Clinical characteristics of CIP in different phases

The acute phase is within the 5^th^ week (range: 1–8^th^ week) after the diagnosis of CIP, the subacute phase is within the 5^th^~13^th^ week (range: 9–19^th^ week), and the chronic phase is after the 13^th^ week (range: 9–19^th^ week). CIP’s most common clinical feature was cough (n=37, 72.5%). From the acute phase to the chronic phase, the incidence of the common symptoms other than cough decreased (shortness of breath: 64.7% vs. 40.9% vs. 27.3%, *P*=0.032; expectoration: 60.8% vs. 50.0% vs. 18.2%, *P*=0.035; fever: 19.6% vs. 0.0% vs. 0.0%, *P*=0.021). The proportion of asymptomatic patients also increased with the CIP development (*P*=0.014). Otherwise, the severity or frequency of symptoms decreased from the acute to chronic phases, as evidenced by the proportion of the severity of cough and shortness of breath. According to clinical evaluation, the acute phase had the highest proportion of patients with high PS score (3~4) and CTCAE grade (45.1% vs. 13.6% vs. 0.0%, *P*=0.001; 47.1% vs. 22.7% vs. 0.0%, *P*=0.003). More information is displayed in **Table 2**.

**Table 2:** Clinical features of patients in different phases.

	Acute phase (N=51)	Subacute phase (N=22)	Chronic phase (N=11)	*P* value
Clinical manifestations – N (%)				
Cough	37 (72.5%)	16 (72.7%)	5 (45.5%)	0.210
Frequent	31 (83.8%)	12 (75.0%)	1 (20.0%)	0.013*
Intermittent	6 (16.2%)	4 (25.0%)	4 (80.0%)	
Shortness of breath	33 (64.7%)	9 (40.9%)	3 (27.3%)	0.032*
At rest	20 (60.6%)	2 (22.2%)	0 (0.0%)	0.025*
After activity	13 (39.4%)	7 (77.8%)	3 (100.0%)	
Expectoration ^a^	31 (60.8%)	11 (50.0%)	2 (18.2%)	0.035*
Fever	10 (19.6%)	0 (0.0%)	0 (0.0%)	0.021*
Asymptomatic	4 (7.8%)	6 (27.3%)	4 (36.4%)	0.014*
ECOG PS score – N (%)				
0~2	28 (54.9%)	19 (86.4%)	11 (100.0%)	0.001**
3~4	23 (45.1%)	3 (13.6%)	0 (0.0%)	
CTCAE grade – N (%)				
1~2	27 (52.9%)	17 (77.3%)	11 (100.0%)	0.003**
3~4	24 (47.1%)	5 (22.7%)	0 (0.0%)	

ECOG PS, Eastern Cooperative Oncology Group Performance Status; CTCAE, Common Terminology Criteria for Adverse Events.

*P<0.05, **P<0.005. ^a^Expectoration with white sputum.

### Radiologic features of CIP in different phases

A total of 51 patients had CT images at the initial phase of CIP. Ground-glass opacities (GGOs) and/or patchy shadows appeared in 48 of 51 (94.1%) CIP patients, with 14.6% of these cases accompanied by air bronchogram. Stride shadow could be seen in 31.4% of patients, and consolidation could be seen in 17.7%. A diffuse nodular lesion appeared in 15.7% of patients. A small number of patients had CT findings showing reticular opacities or traction bronchiectasis (9.8% respectively). Interlobular septal thickening occurred in 19.6% patients.

**Figure 2:**
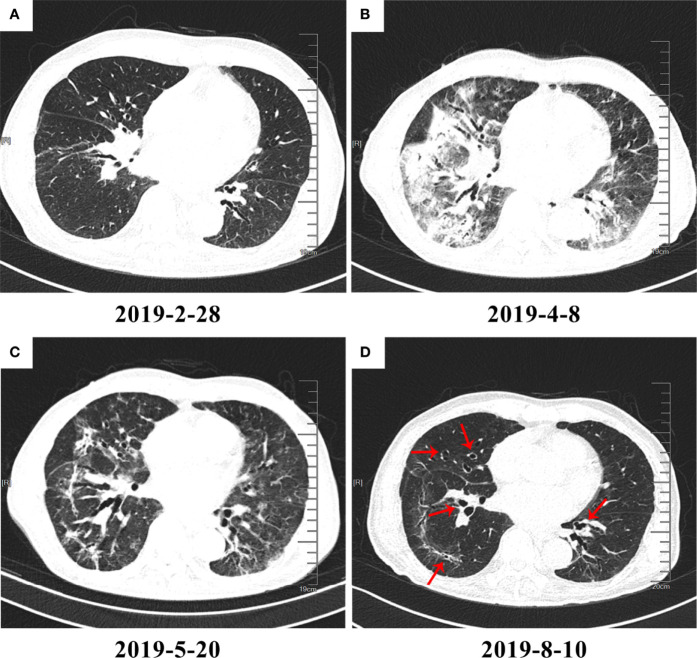
High-resolution computed tomography (HRCT) imaging of checkpoint inhibitor–related pneumonitis (CIP) in different phases. On March 1, 2019, the patient began immunotherapy and developed CIP on April 8, 2019. **(A)** Baseline HRCT image. **(B)** CIP in the acute phase. Diffuse patchy shadows and ground-glass opacities (GGOs) with consolidation can be seen in the image. **(C)** CIP in the subacute phase. The patchy shadows, GGOs, and consolidation appear to have been greatly absorbed. However, traction bronchiectasis can be seen developing to a severe degree. **(D)** CIP in the chronic phase. Most inflammation-related changes have been absorbed, and the lesions are mainly leftover traction bronchiectasis (red arrows).

Fourteen patients who had CT images in at least 2 phases were selected to record the changes in characteristics in different phases. The main change from the acute phase to the chronic phase was the absorption of the CIP lesions, but the characteristics did not change between every 2 phases. In addition to absorption, an increase in chronic or fibrosis–related features such as traction bronchiectasis was observed in 3 patients. An example of typical CT changes from the acute to chronic phases is presented in **Figure 2**.

### Histologic features of CIP in different phases

Fourteen patients (15 slides, with one patient having lung biopsy in 2 phases) underwent tissue biopsy (13 in bronchoscopic biopsy and 2 in ultrasound-guided percutaneous lung puncture) of the CIP lesion: 8 specimens were in the acute phase, 6 in the subacute phase, and 1 in the chronic phase only. Because the chronic phase only had one specimen, proportion statistics were only applied to the acute and subacute phases.

**Figure 3:**
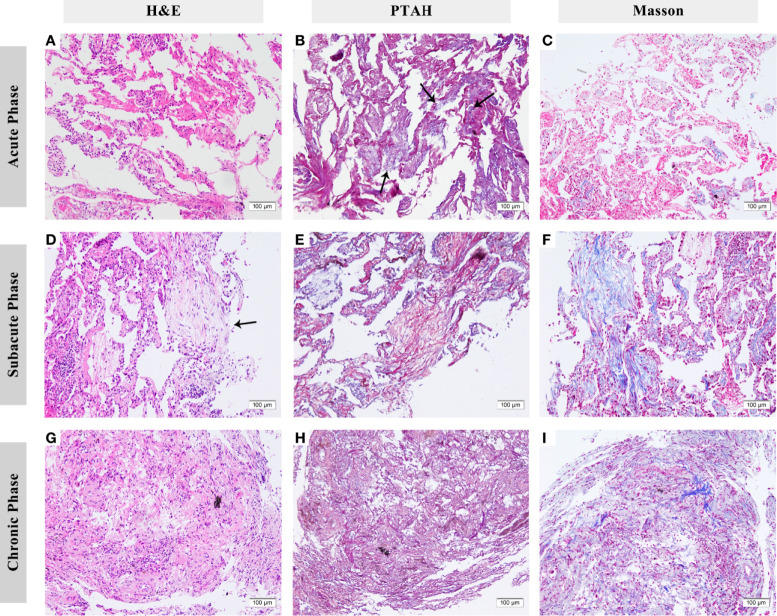
Histologic features of checkpoint inhibitor-related pneumonitis (CIP) in different phases. **(A–C)** Lung biopsy eight days after the onset of CIP. Fibrinous exudation can be seen in the alveolar cavities, especially under phosphotungstic acid hematoxylin (PTAH) staining (arrows). **(D–F)** Lung biopsy 83 days after onset. Organizing changes are dominant (arrows). Under PTAH and Masson staining, it can be seen that the lesion is in a state of coexistence of fibrinous exudation and fibrosis. **(G–I)** Lung biopsy 139 days after onset. The fibrosis replaced the normal structure of alveoli with the thickening of blood vessels. H&E, hematoxylin and eosin. PTAH, phosphotungstic acid hematoxylin.

**Figure 4:**
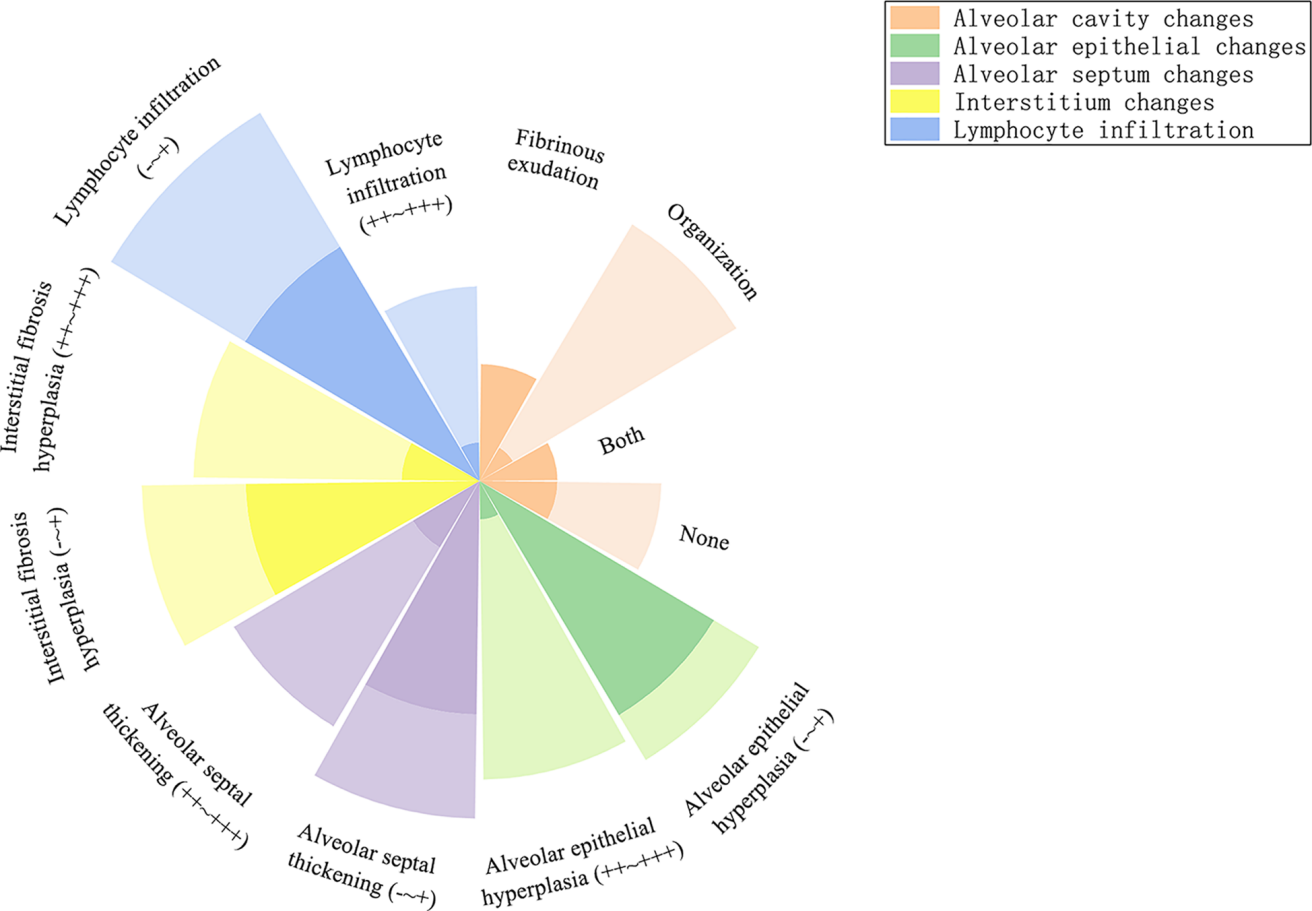
Radial stacked column chart of different histologic features in the acute and subacute phases. The dark part represents the proportion of the number of people with this characteristic to the total population in the acute phase, and the light part represents the subacute phase.

In the acute phase, acute fibrinous pneumonitis was the main histologic pattern (5/8), which was characterized by fibrinous exudation in the alveolar cavities (**Figures 3A–C**) and was accompanied by mild organization formation in 3 of 8 patients. Furthermore, the severity of alveolar epithelial hyperplasia, alveolar septal thickening, interstitial fibrosis, and lymphocyte infiltration was mild (**Figure 4**). In the subacute phase, 5 of 6 patients had the features of organizing pneumonia (**Figures 3D–F**). Other pathologic features were more severe than those in the acute phase (**Figure 4**), and one specimen had mild lymphocyte infiltration. In the chronic phase specimen, the normal lung structure was damaged entirely, with fibrosis with thickening and occlusion of blood vessels being the predominant pathologic changes (**Figures 3G–I**).

### Laboratory examination of CIP in different phases

**Figure 5:**
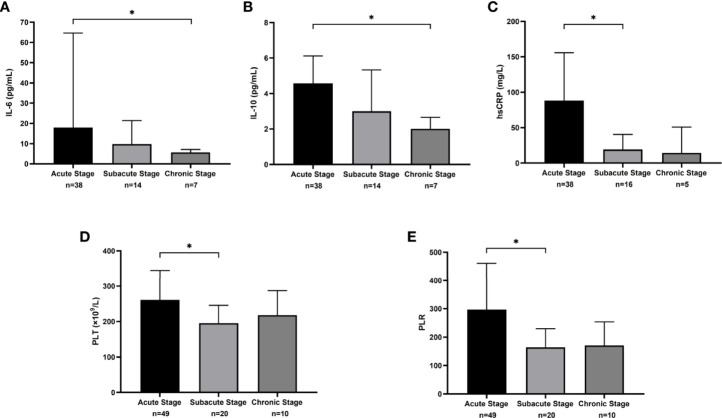
The median and interquartile range of laboratory measures that were statistically different across clinical phases. **(A)** The results of interleukin (IL)-6 (pg/mL). **(B)** The results of IL-10 (pg/mL). **(C)** The results of sensitivity C-reactive protein (hsCRP) (mg/L). **(D)** The results of platelet count (PLT) (×10^9^/L). **(E)** The results of platelet-to-lymphocyte ratio (PLR).

The values of interleukin (IL)-6, IL-10, hsCRP, platelet count (PLT), and platelet-to-lymphocyte ratio (PLR) among the 3 phases showed statistically significant differences. For IL-6 and IL-10, the value decreased with time (17.9 vs. 9.8 vs. 5.7 pg/mL, *P*=0.018; 4.6 vs. 3.0 vs 2.0 pg/mL, *P*=0.041). In pairwise comparisons, only the difference between the acute and chronic phases for IL-6 showed statistical significance (*P*=0.018; **Figures 5A, B**). The value of hsCRP also decreased from the acute phase to the chronic phase (88.2 vs. 19.4 vs. 14.4 mg/L, *P*=0.005). A significant decline in the hsCRP value was observed between the acute and subacute phases (*P*=0.018; **Figure 5C**). For PLT and PLR, the value in the subacute phase was lower than that of the other phases (261.0 vs. 195.5 vs. 218.0 ×10^9^/L, *P*=0.032; 296.7 vs. 164.1 vs. 170.7, *P*=0.013) and was significantly different between the acute phase and the subacute phase (*P*=0.046 and *P*=0.047; **Figures 5D, E**). The results of other laboratory examinations are summarized in **e-Table 1**.

Based on the above results, we summarized the characteristics of CIP in different phases in **Table 3**.

**Table 3:** Features of checkpoint inhibitor-related pneumonitis (CIP) in different phases.

Phase	Timing^a^	Clinical features	Radiologic features	Histologic features	Laboratory test	Prognosis
Acute	≤5 weeks	Cough/expectoration/shortness of breath/fever. More severe and with high onset frequency.	GGO/patchy shadows/stride shadow/consolidation/diffuse nodule lesion, accompanying reticular opacities/traction bronchiectasis/interlobular septal thickening.	Fibrinous exudation, with or without organization; mild alveolar epithelial hyperplasia, alveolar septal thickening, interstitial fibrosis hyperplasia, lymphocyte infiltration.	IL-6 ↑ IL-10 ↑ hsCRP ↑	Most patients can obtain symptom relief within a few days, and lesions on imaging appear to be absorbed in a short time.
Subacute	5~13 weeks	Symptoms in the acute phase are relieved with a decrease in frequency.	Features in the acute phase are absorbed. Chronic lesions are developing in some patients.	Organization, with or without fibrinous exudation, moderate or severe alveolar epithelial hyperplasia, alveolar septal thickening, interstitial fibrotic hyperplasia, lymphocyte infiltration.	Compared to the acute phase: IL-6 ↓ IL-10 ↓ hsCRP ↓↓	The improvement rate of symptoms and lesions is slower.
Chronic	≥13 weeks	Cough/expectoration occasionally/shortness of breath after activity. More patients are asymptomatic. Nearly all patients have a low PS score and CIP grade.	Most of the inflammation lesions have been absorbed or remain in a stable state. Some patients have persistent chronic lesions.	Fibrosis with or without thickening of the blood vessel walls and blockage of blood vessels.	The elevated inflammation indicators return to normal, and other laboratory test results indicate a better condition.	The symptoms and lesions remain in a stable state, and further improvement does not generally occur.

a The timing is counted from the appearance of checkpoint inhibitor–related (CIP) symptoms or imaging prompts of CIP. If there is a recurrence of CIP during the process, the new lesions are considered to be in the acute phase. ↑Numerical increase. ↓Numerical decrease.

CIP, checkpoint-inhibitor pneumonitis; GGO, ground-glass opacity; IL-6, interleukin-6; IL-10, interleukin-10; hsCRP, high sensitivity C-reactive protein; PS, performance status.

## Discussion

The recent emergence of CIP and its negative impact on the efficacy of ICIs have garnered greater research attention, as is the case with the treatment of irAEs. With in-depth study, the different potential mechanisms of different irAEs have been proposed, which has led to the greater personalization of the management of irAEs (20). Tailored clinical management should be applied not only across different irAEs but also due to microenvironment changes during the disease, personalized management should be applied during the evolution of a specific irAE as well, meaning that the clarification of the CIP process is critical. In this retrospective study, we attempted to propose new phases of CIP to facilitate personalized treatment.

The symptoms were more severe for clinical presentation in the earlier phases, and the PS score and CIP grade were higher. Although the radiographic patterns of CIP can be categorized into different types (21), the main change of the lesions in this study was absorption regardless of type, and only some cases remained in traction bronchiectasis, meaning a fibrotic change of pulmonary tissue. The results of histology also showed a development from inflammation to fibrosis. Therefore, most CIP patients in this study experienced a change from active inflammation to a steady state with gradual regression of inflammation, and mild fibrosis was the endpoint in some patients. The reason why CIP rarely develops into an irreversible fibrotic state may relate to the mechanisms of CIP. Postow et al. suggest four potential mechanisms of irAEs (1): activated T-cells against antigens presenting in tumor and healthy tissue (2); increase in the level of inflammatory cytokines (3); preexisting autoantibodies (4); direct binding of ICIs to normal tissue (22). According to Zhai et al. (23), since PD-1/PD-L1 is mainly expressed in immune cells, with no indication of high expression in normal lung tissues, the mechanisms of CIP may be more related to the first three theories. In other words, the mechanisms of CIP are potentially more related to immune disorders than to directly irreversible damage caused by treatments like radiotherapy.

The concept of chronic CIP has been mentioned in several articles. A multicenter study by John et al. in 2019 found that 64% of CIP patients had incomplete absorption of lung CT lesions at imaging follow-up of more than six months. Although most patients had resolution of clinical symptoms, frequent persistence of scarring, nodularity, and ground glass opacities existed (24). This result indicates that many CIP patients maintain a chronic persistent disease-carrying state after partial absorption of the lesions. Chronic irAEs were defined as those extending 12 or more weeks past treatment discontinuation, though this concept has not met a consensus (25, 26). Naidoo et al. defined chronic CIP as pneumonitis that persists or worsens with steroid tapering and necessitates ≥12 weeks of immunosuppression after ICI discontinuation (27). Although the definitions in different articles are slightly different, the time to enter the chronic phase is defined as 12 weeks, which is similar to the 13 weeks obtained in our study. Moreover, we comprehensively analyzed the symptoms, imaging, and laboratory examinations in different periods based on this time, which provided more reference indicators for the staging of CIP, which provided a particular reference value for the subsequent precise treatment of CIP.

For most CIP patients, inflammation was predominant in lesions in the acute phase, and patients exhibited absorption or fibrosis after comprehensive management. The relationship between inflammation and fibrosis has been investigated for some time (28). Fibrosis originates from an excess of the body’s wound-healing mechanisms, and one of the essential triggers is inflammation. The mechanisms of fibrosis are associated with many kinds of cells and key mediators, such as fibroblasts, endothelial cells, and cytokines. Although the mechanisms seem organ-independent, studies have shown that the dominant factors of fibrosis vary across different organs (29). In our results, the level of IL-6, IL-10, and hsCRP increased when CIP occurred and decreased during the relief process of CIP. All 3 of these indexes are related to inflammation: IL-6 is a member of the proinflammatory cytokine family, IL-10 has potent anti-inflammatory properties, and hsCRP is a marker of inflammation and the downstream molecular product of IL‐6. In a previous case report, an increase in the level of IL-6 and IL-10 was found in CIP (30). The changes in these indicators indicate the gradual subsiding of inflammation from the acute phase to the chronic phase. The profibrotic effects of IL-6 in pulmonary fibrosis have also been observed (31, 32), which suggests the potential contribution of IL-6 in the fibrotic changes in the chronic phase of CIP. Moreover, several studies have demonstrated elevated levels of IL-17, IL-35, and Th1/Th17 cells in both peripheral blood and bronchoalveolar lavage fluid in CIP (33, 34). These indicators also have direct or indirect anti- or profibrotic functions (35). Due to the limited clinical testing data, fewer relevant laboratory indicators were examined in our study. More translational studies are needed to explore the relevant mechanisms of the occurrence and development of CIP (20).

Glucocorticoids are currently the first-line treatment for CIP. However, its application still has many uncertainties, such as the total length of use, the time and speed of drug tapering, and the balance between efficacy and adverse effects. In clinical application, infection caused by long-term use of glucocorticoids is common. In our study, one patient underwent long-term oral methylprednisolone treatment (16 mg daily) to facilitate complete absorption of the remaining lesions and prevent a recurrence. After nine months of glucocorticoid treatment, a fungal infection occurred, which threatened the stability of the patient’s condition (the HRCT is shown in **Supplemental Figure E1**). The anti-inflammatory and immunosuppressive mechanisms of glucocorticoids affect all immune cells virtually. The use of glucocorticoids may impair opsonization and phagocytic function, T-cell migration and proliferation, and eosinophilic proliferation, all of which can increase the risk of pathogenic infection (36). A population-based cohort study that included over 275,072 adults found that the risk of respiratory tract infection was higher in glucocorticoid-exposed patients than in those not exposed to glucocorticoids (37). Moreover, higher doses (≥20 mg prednisone-equivalent dose daily) and longer duration (≥4–8 weeks) of glucocorticoids are two factors associated with an increased risk for infection (36). Some studies have reported that even low-dose glucocorticoids can raise the risk of infection (38, 39). Given the information above, along with the experiences acquired in the management of pulmonary fibrosis and radiation pulmonary fibrosis (15, 40), glucocorticoids should be avoided in the chronic phase to reduce unwanted adverse events. Moreover, the protracted condition of some CIP patients is related to the tumor progression or their underlying diseases or complications. Therefore, for patients in the chronic phase, greater attention should be paid to antitumor, anti-infection, and other supportive treatments rather than the long-term use of glucocorticoids. We also believe that the statistical duration of each phase observed in this study can provide more informed guidance for the application duration of glucocorticoids.

Because fibrosis occurred in some of the CIP patients studied, antifibrotic treatment may help prevent and/or slow down the fibrotic process. Although there is no effective treatment to reverse fibrosis, nintedanib and pirfenidone can help to retard its progression (41, 42). Additionally, the anti-angiogenic effects of nintedanib, along with anti-inflammatory and antioxidant properties of pirfenidone, are well-known (43) and may bring similar benefits to CIP patients. Currently, 3 case reports have shown that nintedanib/pirfenidone can bring benefits to CIP (44–46). Furthermore, an open-label study with pirfenidone on chronic hypersensitivity pneumonitis, a kind of immune-related pneumonitis, suggests that adding pirfenidone to anti-inflammatory treatment may improve outcome (47). Furthermore, these two drugs may enhance the efficacy of antitumor therapy (48–50). Therefore, we suggest adding them to CIP treatment, especially before or at the incipience of the chronic phase. However, as the evidence for the use of antifibrotic treatment in CIP is lacking, prospective, randomized clinical trials are needed to assess the real impact of antifibrotic therapy in CIP.

The results of this study should be carefully evaluated before drawing definitive conclusions. First, this is a multi-center retrospective study which may bring bias, such as differences in the testing methods of each center. Second, the clinical phasing in this study requires further refinement, and the clinical manifestations of CIP vary widely. For example, the different diagnosis types in radiology may impact the duration and prognosis of CIP, whether radiomics is better than only HRCT or not. Third, the sample size in the chronic phase and the patients who received tissue biopsy was small. Future studies with larger sample sizes need to further verify the differences among phases. Moreover, some tests, such as immunohistochemistry, lung function test, and bronchoalveolar lavage fluid test, were not employed, and thus the more specific mechanisms in different phases could not be investigated. Research into other facets of CIP should be conducted in the future to establish a more precise clinical phasing of CIP and apply it in the clinic.

**Figure 6:**
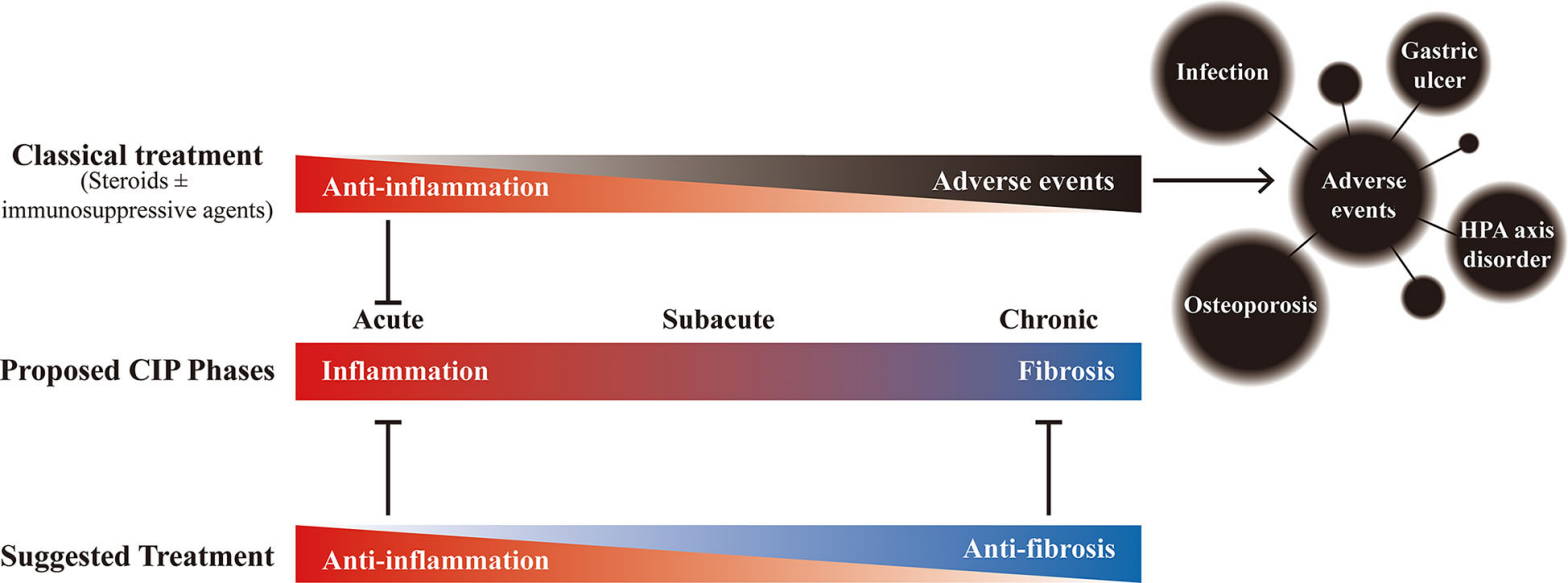
The proposed phases of checkpoint inhibitor-related pneumonitis (CIP) and the effects of different treatments. Under the potential development process of CIP, although the classic treatment (steroids ± immunosuppressive agents (such as infliximab and mycophenolate mofetil)) can bring anti-inflammatory effect in the early stage, long-term use not only fails to bring benefits but also may bring adverse events that affect the overall prognosis. Therefore, it is recommended to gradually transition to anti-fibrotic therapy after adequate anti-inflammatory treatment to achieve appropriate management of the whole process of CIP.

Based on the comprehensive analysis of timing, clinical characteristics, radiologic features, histologic features, and laboratory values of CIP, the development of CIP can be divided into 3 phases: acute, subacute, and chronic. According to the features present from inflammation to fibrosis, the personalized management of these different phases might be devised (**Figure 6**). But the generalizability of this conclusion is limited, and further verification and a larger sample size are needed in the future study.

## Data availability statement

The raw data supporting the conclusions of this article will be made available by the authors, without undue reservation.

## Ethics statement

Written informed consent was obtained from the individual(s) for the publication of any potentially identifiable images or data included in this article.

## Author contributions

CZZ, YY, and XL, and NF were involved in the original conception and design of the work. HD, MW, GQ, and NS were involved in the collection of raw data. YY, LC, JJ, and YD performed data analysis and interpretation. CZZ, YY, and XL drafted the manuscript. RC and YS is the guarantor for this paper. All authors contributed to the revision of data and manuscript. All authors read and approved the final manuscript.

## Funding

This work is supported by Zhongnanshan Medical Foundation of Guangdong Province [no. ZNSA-2020003], the Guangdong Science and Technology Program Special Projects [2020A1111350025], and the State Key Laboratory of Respiratory Disease - The Independent project [SKLRD-Z-202117].

## Conflict of interest

Dr. YK grants or contracts from Systematic Review Workshop Peer Support Group (not for profit organization) and Yasuda Memorial Medical Foundation. Dr. SW receives honoraria for lectures from AstraZeneca, Chugai Pharma, Ono Pharmaceutical, Bristol-Myers, Boehringer Ingelheim, Eli Lilly, MSD, Taiho Pharmaceutical, Pfizer, Novartis and Daiichi Sankyo.

The remaining authors declare that the research was conducted in the absence of any commercial or financial relationships that could be construed as a potential conflict of interest.

## Publisher’s note

All claims expressed in this article are solely those of the authors and do not necessarily represent those of their affiliated organizations, or those of the publisher, the editors and the reviewers. Any product that may be evaluated in this article, or claim that may be made by its manufacturer, is not guaranteed or endorsed by the publisher.

## Abbreviations

CIP, checkpoint inhibitor-related pneumonitis; CTCAE, common terminology criteria for adverse events; GGO, ground-glass opacities; H&E, hematoxylin and eosin; HP, hypersensitivity pneumonitis; HRCT, high-resolution computed tomography; hsCRP, sensitivity C-reactive protein; ICIs, immune checkpoint inhibitors; IL, interleukin; irAEs, immune-related adverse events; PD-1/PD-L1, programmed cell death protein 1/programmed death ligand 1; PLR, platelet-to-lymphocyte ratio; PLT, platelet count; RILI, radiation-induced lung injury.
