# Supplementary material for: Proposed clinical phases for the improvement of personalized treatment of checkpoint inhibitor–related pneumonitis
Source: Front Immunol. 2022 Jul 20;13:935779. doi: 10.3389/fimmu.2022.935779 (PMC9364904; doi:10.3389/fimmu.2022.935779)
Supplement: Supplementary file 1 [file DataSheet_1.docx]

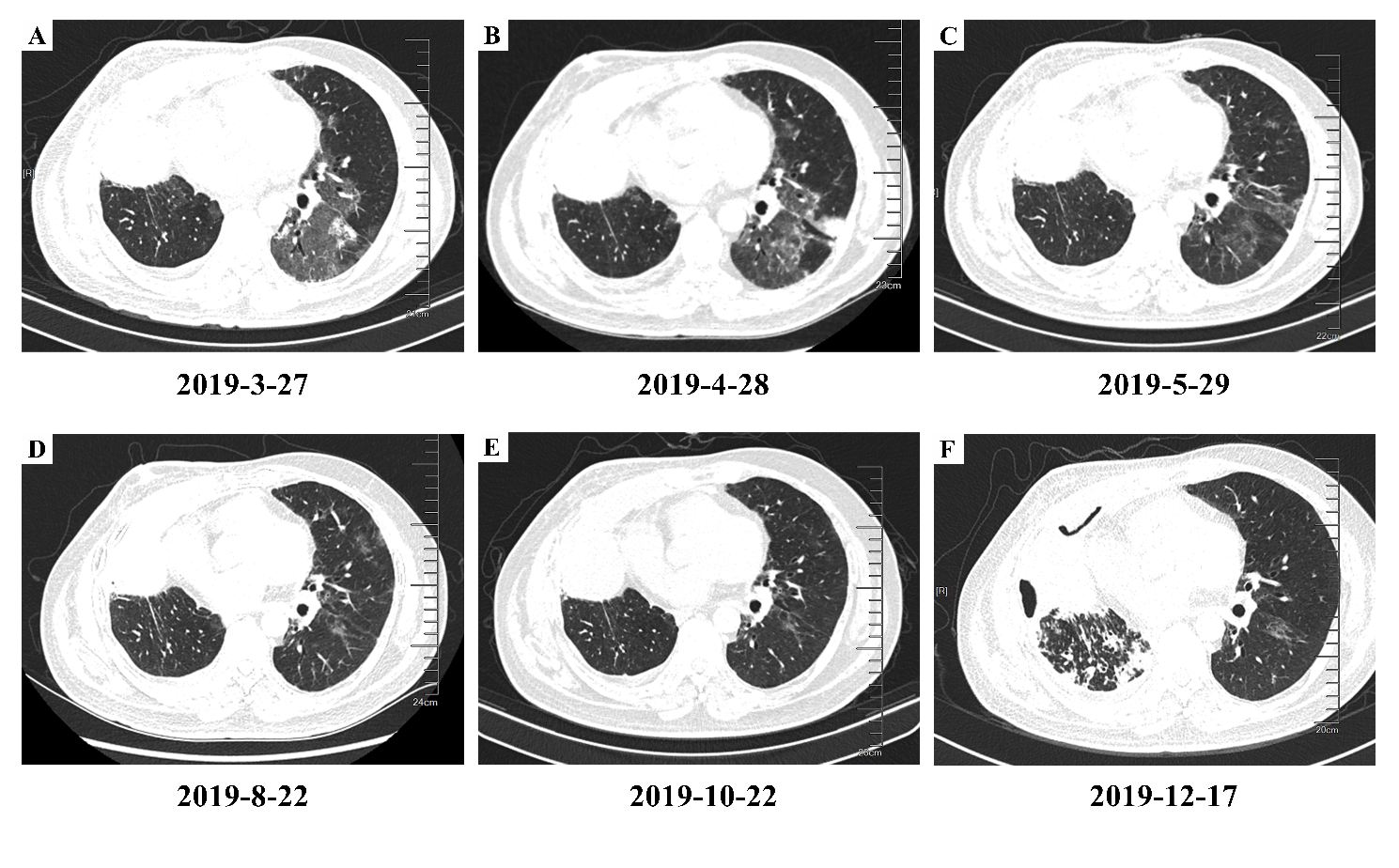


**e-Figure 1** **High-resolution computed tomography (HRCT) of a patient who had a fungal infection after long-term administration of glucocorticoids** This patient had checkpoint inhibitor–related pneumonitis (CIP) 8 months after immune checkpoint inhibitor treatment. After treatment with methylprednisolone 40 mg iv qd, the symptoms were relieved. The regimen was gradually decreased to 16 mg methylprednisolone po qd, which continued in this manner until December 2019. The patient restarted immunotherapy in August 2019, and continuous HRCT monitoring did not detect significant recurrence of CIP. Beginning in November 2019, the patient had obvious cough and expectoration, accompanied by shortness of breath after activity. In December, the patient returned to the hospital for follow-up, and the examination results suggested a fungal infection.
