# Supplementary material for: Proposed clinical phases for the improvement of personalized treatment of checkpoint inhibitor–related pneumonitis
Source: Front Immunol. 2022 Jul 20;13:935779. doi: 10.3389/fimmu.2022.935779 (PMC9364904; doi:10.3389/fimmu.2022.935779)
Supplement: Supplementary file 2 [file Table_1.docx]

**e-Table 1 Laboratory test of patients in different phases.**

|  | Acute phase | Subacute phase | Chronic phase | *P* value |
| --- | --- | --- | --- | --- |
| Routine blood test – median (interquartile range)  WBC – (×10^9^/L)  NEUT – (×10^9^/L)  LYMPH – (×10^9^/L)  MONO – (×10^9^/L)  EO – (×10^9^/L)  PLT – (×10^9^/L)  NLR  PLR | 7.8 (5.9)  6.0 (6.0)  1.0 (1.0)  0.7 (0.5)  0.1 (0.3)  261.0 (162.0)  6.6 (8.1)  296.7 (314.45) | 8.1 (4.1)  5.6 (5.1)  1.2 (0.8)  0.6 (0.4)  0.1 (0.2)  195.5 (87.5)  4.2 (9.2)  164.1 (105.7) | 6.0 (4.5)  4.4 (1.9)  1.0 (1.2)  0.7 (0.4)  0.1 (0.3)  218.0 (150.3)  3.7 (3.4)  170.7 (159.8) | 0.228  0.141  0.417  0.452  0.311  0.032*  0.138  0.013* |
| Cytokines – median (interquartile range)  IL-2 – (pg/mL)  IL-4 – (pg/mL)  IL-6 – (pg/mL)  IL-10 – (pg/mL)  TNF-α – (pg/mL)  IFN-γ – (pg/mL) | 1.0 (1.0)  1.5 (1.7)  17.9 (57.0)  4.6 (3.8)  1.2 (1.6)  1.6 (2.1) | 1.0 (1.9)  1.6 (1.4)  9.8 (17.4)  3.0 (3.8)  1.2 (1.7)  0.8 (1.6) | 1.0 (1.2)  1.2 (1.2)  5.7 (4.2)  2.0 (1.0)  1.1 (1.9)  1.2 (1.7) | 0.785  0.868  0.018*  0.041*  0.777  0.071 |
| KL-6 – median (interquartile range) (U/mL) | 472.0 (1546.0) | 557.5 (1832.5) | 451.0 (689.3) | 0.537 |
| Liver function – median (interquartile range)  ALB – g/L  GGT – U/L | 33.5 (7.1)  49.2 (71.9) | 35.1 (4.2)  53.6 (53.3) | 35.1 (8.0)  84.6 (103.2) | 0.121  0.803 |
| Biochemical index – median (interquartile range)  LDH – U/L  Inflammatory biomarkers – median (interquartile range)  hsCRP – mg/L | 249.5 (115.6)  88.2 (138.0) | 210.3 (156.1)  19.4 (37.0) | 240.0 (173.1)  14.4 (47.9) | 0.847  0.005** |
| PCT increase – N (%) | 21 (87.5%) | 5 (55.6%) | 3 (75.0%) | 0.135 |

* *P*＜0.05. ** *P*＜0.01

WBC, white blood cell count. NEUT, neutrophil count. LYMPH, lymphocyte count. MONO, monocyte count. EO, eosinophil count. PLT, platelet count. NLR, neutrophil-to-lymphocyte ratio. PLR, platelet-to-lymphocyte ratio. IL-2, interleukin-2. IL-4, interleukin-4. IL-6, interleukin-6. IL-10, interleukin-10. TNF-α, tumor necrosis factor α. IFN-γ, interferon-γ. KL-6, krebs von den lungen-6. ALB, albumin. GGT, gamma-glutamyl transferase. LDH, lactate dehydrogenase. hsCRP, sensitivity C-reactive protein. PCT, procalcitonin. N, number of cases.
